# Quantitative determination of spin–orbit-induced magnetic field in GaMnAs by field-scan planar Hall measurements

**DOI:** 10.1038/s41598-021-89748-6

**Published:** 2021-05-13

**Authors:** Seongjoon Park, Shinwoo Lee, Kyung Jae Lee, SeongJin Park, Phunvira Chongthanaphisut, Jiyeong Jang, Sanghoon Lee, Xinyu Liu, M. Dobrowolska, Jacek K. Furdyna

**Affiliations:** 1 Physics Department, Korea University, Seoul, 136-701 Republic of Korea; 2 Physics Department, University of Notre Dame, Notre Dame, IN 46556 USA

**Keywords:** Physics, Condensed-matter physics, Spintronics

## Abstract

Spin–orbit-induced (SOI) effective magnetic field in GaMnAs film with in-plane magnetic anisotropy has been investigated by planar Hall effect measurements. The presence of SOI field was identified by a shift between planar Hall resistance (PHR) hystereses observed with positive and negative currents. The difference of switching fields occurring between the two current polarities, which is determined by the strength of the SOI field, is shown to depend on the external field direction. In this paper we have developed a method for obtaining the magnitude of the SOI fields based on magnetic free energy that includes the effects of magnetic anisotropy and the SOI field. Using this approach, the SOI field for a given current density was accurately obtained by fitting to the observed dependence of the switching fields on the applied field directions. Values of the SOI field obtained with field scan PHR measurements give results that are consistent with those obtained by analyzing the angular dependence of PHR, indicating the reliability of the field scan PHR method for quantifying the SOI-field in GaMnAs films. The magnitude of the SOI field systematically increases with increasing current density, demonstrating the usefulness of SOI fields for manipulation of magnetization by current in GaMnAs films.

## Introduction

Recently, spin–orbit-torque (SOT) effects, in which spin-angular momentum transfers from spin-polarized carriers to local magnetic moments, has become a key technique for manipulation of magnetization in spintronic applications^1–5^. In this process the required spin current is normally generated in a heavy metal (HM) by the spin Hall effect along the direction perpendicular to the current. To use such spin current for manipulating magnetization, one often uses structures in the form of ferromagnetic (FM)/HM bilayers, in which propagation of spin current from the HM to the FM exerts a spin–orbit torque (SOT) on the magnetization of the FM layer. In FM/HM bilayer systems, however, structural inversion symmetry is broken at the interface, allowing a spin polarization of carriers, with same symmetry as spin Hall effect, to also be generated by the Rashba effect^6,7^. Such polarized carriers can then also exert SOT on the magnetization of the FM layer. Although a large number of SOT studies has already been carried out on FM/HM bilayer systems, the mechanisms for SOT are still under investigation, with an eye on finding more effective ways of controlling magnetization by an applied current^8–11^.

In addition to the spin Hall and the Rashba effect, it is known that in crystalline systems with broken bulk inversion symmetry the spin polarization of carriers can also be achieved by the Dresselhaus effect^12^. This form of spin polarization of carriers can then also exert a SOT on local magnetic moments, and thus can be used to manipulate the magnetization. For the utilization of this mechanism, a magnetic crystalline film with broken bulk inversion symmetry is required. One of the best materials systems satisfying this requirement is a crystalline ferromagnetic semiconductor GaMnAs, in which bulk inversion symmetry is broken by zinc-blende structure and ferromagnetism is realized by incorporating Mn ions in the system^13–15^. In a GaMnAs system, moving carriers are spin-polarized by spin–orbit-induced (SOI) effective magnetic field, and thus they exert a SOT on the local magnetic moments.

Furthermore, when the GaMnAs films are grown on GaAs substrates, the strain between the adjacent layers also causes an effective magnetic field in the GaMnAs film with the same symmetry as the Dresselhaus effect^16^. Such strain-induced effective field is indeed dominant in the GaMnAs film^17^. Since the strain-induced spin–orbit field and the effective field induced by the Dresselhaus effect have the same symmetry, the two effective fields are jointly referred to the Dresselhaus-type SOI field.

The presence of the Dresselhaus-type SOI field and SOT manipulation of magnetization in a single crystalline ferromagnetic film was first observed in GaMnAs film^16^. Further investigations show that the current density used for the manipulation of magnetization in GaMnAs films is in the range of 10^5^–10^6^ A/cm^2^^16–20^, which is one or two orders of magnitude smaller than that used in FM/HM bilayer systems^5,21^. This points to the importance of using the Dresselhaus-type SOI field in current manipulation of magnetism, and calls for detailed quantitative investigation of the SOI field in GaMnAs films.

A highly effective method for studying SOI fields is to use Hall resistance measurements. For example, second harmonic Hall voltage measurements are commonly used to study SOI fields^22,23^. In standard DC Hall measurements, the SOI field in a GaMnAs film was first quantified in the work of Chernyshov et al. published in Nature Physics^16^. In that work, the SOI field was determined by considering a geometrical construction that involves the applied magnetic field and the difference in magnetization switching angles. Later, a new method was developed by considering magnetic free energy based on domain nucleation and propagation model for that occurs in magnetization reversal process observed by rotating a magnetic field^24^. However, field scan measurements are much more commonly used for studying the process of magnetization reversal, and it is therefore desirable to use such measurements to study the SOI field. There exists only one article, which reports a quanttative study of SOI fields in GaMnAs films by using field scan measurement^25^. However, in that work the authors use a coherent rotation model in the analysis, which only be applied at large external magnetic fields. Since the magnetization reversal in GaMnAs film is known to occur via domain nucleation and propagation process at relatively weak fields, applying the coherent rotation model to fit field-scan data, including magnetization transitions during magnetization reversal, results in significant error. In the present manuscript we have therefore developed a new method of analyzing field scan data, based on a domain nucleation and propagation model for magnetization reversal, which is applicable at relatively weak fields. This analysis only deals with switching fields that occur in the low field region, without considering the behavior at large fields. The model describes the magnetization reversal process more accurately, thus providing the values of SOI fields with significantly less uncertainty. This investigation provides a convenient and accurate method for quantifying the SOI fields by PHR measurements, complementary to angle scan measurements used in earlier studies.

## Experiment

**Figure 1:**
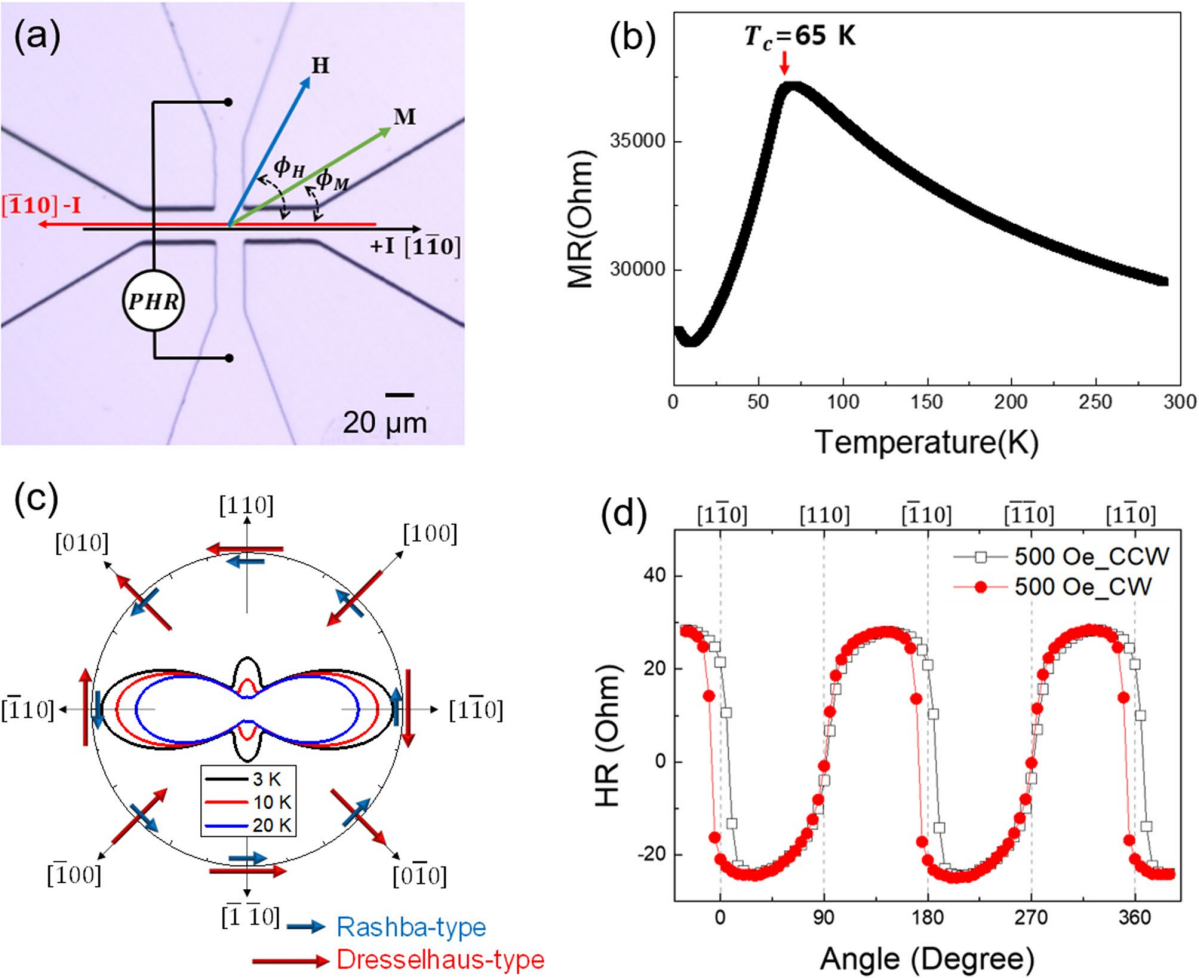
(**a**) Microscopic image of Hall device patterned on GaMnAs sample. Directions of current, external magnetic field, and magnetization are shown by arrows. (**b**) Temperature dependence of resistance for GaMnAs film at zero magnetic field. The signature of ferromagnetic transition appears as a peak near 65 K. (**c**) Polar plot of magnetic free energy density for our GaMnAs film obtained at three different temperatures. Directions of Rashba-type and Dresselhaus-type SOI fields are shown by thick blue and red arrows, respectively, for current directions indicated by thin black arrows. (**d**) Angular dependence of planar Hall resistance obtained by rotating a field of 500 Oe at 3 K. The open (black) squares and solid (red) circles show data obtained by CCW and CW rotations of the field in the film plane, respectively.

A layer of GaMnAs was grown on a (001) GaAs substrate by low temperature (LT) molecular beam epitaxy (MBE). After deoxidation of the GaAs surface, a 250 nm GaAs buffer layer was first grown at 600 °C. Then an additional 2 nm LT-GaAs buffer layer was deposited at 250 °C. At the same substrate temperature, a Ga_1*-x*_Mn_*x*_As layer with *x* = 0.05 was grown to a thickness of 40 nm. For transport measurements, we fabricated a crossbar Hall device shown in Fig. 1a using photolithography and chemical etching. The $\left[ {1\overline{1}0} \right]$ and the $\left[ {\overline{1}10} \right]$ crystallographic directions and the measurement scheme are shown in the figures. The electromagnet used for the PHR measurements was mounted on a rotating table, allowing us to control the field direction in the film plane. The direction of the external magnetic field $\varphi_{H}$ and magnetization $\varphi_{M}$ used in discussing the PHR data are measured from the $\left[ {1\overline{1}0} \right]$ crystalline direction, which is also the positive direction of the current. The Curie temperature of the GaMnAs film was estimated from the resistance peak shown in Fig. 1b to be 65 K^26^. The magnetic anisotropy of the GaMnAs film was identified from the angular dependence of the PHR data shown in Fig. 1d, which reveal both the cubic anisotropy along the < 100 > directions and the uniaxial anisotropy along [110]^27,28^. Precise values of magnetic anisotropy parameters were obtained by analyzing the angular dependence of PHR based on magnetic free energy minima conditions^29,30^. The free energy diagrams for three temperatures are plotted in Fig. 1c, showing a systematic transition of anisotropy from the dominant cubic to uniaxial as the temperature increased. In Fig. 1c, the Rashba-type and the Dresselhaus-type SOI fields are shown by blue and red arrows, respectively, for current directions indicated by thin black arrows.

## Results

**Figure 2:**
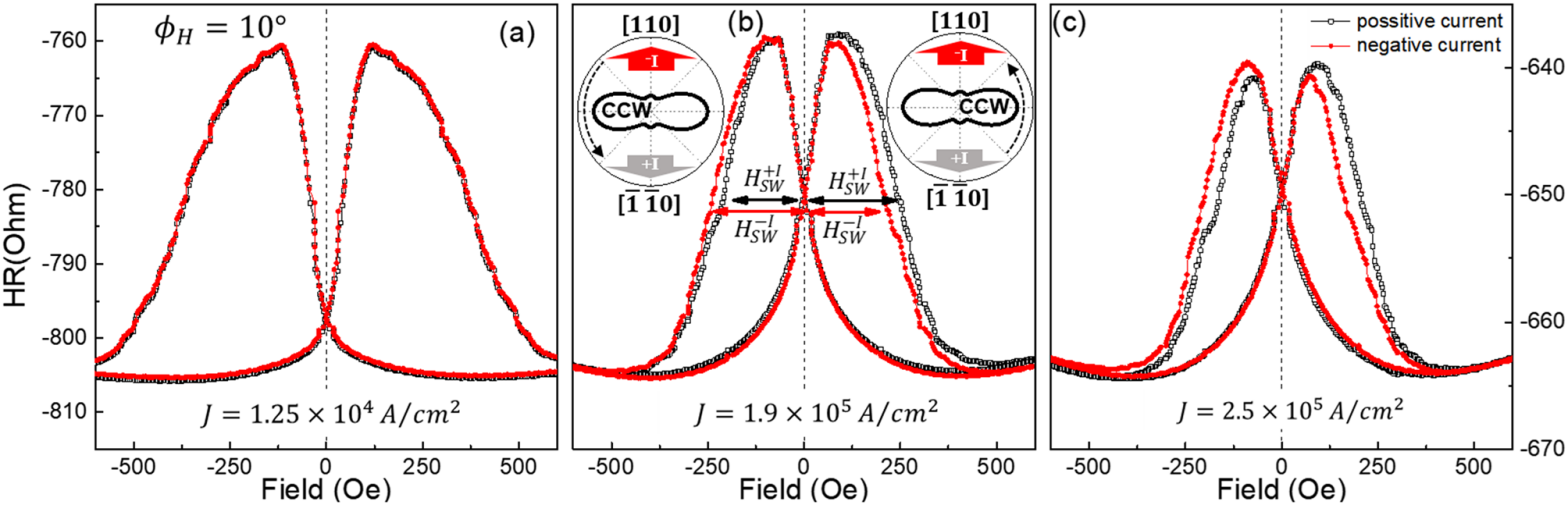
Field scans of PHR measured at 3 K in field scans at $\varphi_{H}$ = 10 $^\circ$ for three different current densities. Black open and red solid symbols represent data measured with positive and negative currents, respectively. The insets in (**b**) show CCW rotation of magnetization over the $\left[ {\overline{1}10} \right]$ and the $\left[ {1\overline{1}0} \right]$ energy barrier in negative and positive field regions, respectively. The thick red and gray arrows in the insets indicate the direction of the SOI field for negative and positive currents, respectively. The shifts of the hysteresis between results for positive and negative currents increase as current density increases.

The presence of SOI field in the GaMnAs film was detected by measuring the planar Hall resistance (PHR) during magnetization reversal. Figure 2 shows the PHR data obtained during a field scan between ± 2000 Oe at 3 K. The direction of the magnetic field for this scan was set at $\varphi_{H} =$ 10°. The data in the three panels of Fig. 2 are obtained at different current densities. Black open and red solid symbols represent PHR data obtained with positive and negative currents, respectively. The behavior of PHR during magnetization reversal is described by^31,32^

1 $$ R_{PHR} = \frac{k}{t}M^{2} {\text{sin}}2\varphi_{M} , $$

where *t* is the film thickness, *k* is a constant related to the anisotropic magnetoresistance (AMR) of the GaMnAs film, and *M* is the magnetization of the film. Although the overall behavior of the PHR hysteresis taken with three current densities looks similar, one can recognize that there is a different feature in the three sets of data. While the PHR hysteresis measured with positive and negative current directions completely overlap for a current density of 1.25 × 10^4^ A/cm^2^ (see Fig. 2a), one can see that the hysteresis curves measured with opposite current polarities gradually shift away from each other as the current density increases to 1.9 × 10^5^ A/cm^2^ and 2.5 × 10^5^ A/cm^2^, as shown in Fig. 2b, c. Such a shift of the hysteresis in opposite direction observed for opposite current polarities indicates the presence of a SOI field in the GaMnAs film, as has been observed in an earlier study^33^.

Note further that the PHR hysteresis systematically shifts to the right for the positive current, and to the left for the negative current. The direction of the shift reflects the direction of the effective SOI field in the GaMnAs film. When the applied field is scanned along $\varphi_{H}$ = 10°, the magnetization of the GaMnAs film makes transitions from the 2nd quadrant to the 3rd over the energy barrier at the $\left[ {\overline{1}10} \right]$ direction in the negative field region, and from the 4th quadrant to the 1st over the $\left[ {1\overline{1}0} \right]$ energy barrier in the positive field regions, as shown in the insets in Fig. 2b. The switching fields corresponding to these transitions are marked as $H_{SW}^{ + I}$ and $H_{SW}^{ - I}$, respectively, for positive and negative currents in Fig. 2b. Even though magnetization switching is not abrupt due to pinning field distribution of domains^34^, using the field at half-peak value provides a good estimate of the average value of switching fields in the film. Note that in the negative field region the magnitude of $H_{SW}^{ + I}$ is smaller than the $H_{SW}^{ - I}$, indicating that the positive current assists the CCW rotation of magnetization over the $\left[ {\overline{1}10} \right]$ energy barrier, while the negative current hinders the same transition. The situation is opposite in the positive field region, where $H_{SW}^{ + I}$ is larger than $H_{SW}^{ - I}$. Such opposite behavior of switching fields can be observed when the effective SOI field points along the $\left[ {\overline{1}\overline{1}0} \right]$ direction in the presence of a positive current, and along [110] when the current is negative, as shown in the insets of Fig. 2b. As seen in Fig. 1c, in the present experimental configuration we only measure the combined effect of Dresselhaus-type and Rashba-type-SOI fields. These effective SOI field directions are consistent with Dresselhaus-type SOI fields shown in Fig. 1c.

Since the effect of the SOI-field on the reorientation of magnetization is appeared as the difference between $H_{SW}^{ + I}$ and $H_{SW}^{ - I}$, this measurement can be used to determine the magnitude of the SOI field in the GaMnAs film. Specifically, the switching fields occurring during magnetization reversal are sensitive functions of the scanning field orientation, and—as will be shown—this dependence can be used to obtain the SOI values. The magnetization reversal processes are analyzed based on magnetic free energy^24,29^, which is given by

2 $$ \frac{E}{M} = \left( {K_{C} /4M} \right)\cos^{2} 2\varphi_{M} + \left( {K_{U} /M} \right)\sin^{2} \varphi_{M} - H\cos \left( {\varphi_{M} - \varphi_{H} } \right) - H_{{{\text{eff}}}} \cos \left( {\varphi_{M} - \varphi_{{{\text{eff}}}} } \right), $$

where $K_{U}$ and $K_{C}$ are uniaxial and the cubic anisotropy coefficients, respectively. The effective SOI field and external magnetic field are expressed as $H_{{{\text{eff}}}}$ and $H$, respectively. The orientations of magnetization $\varphi_{M}$, external field $\varphi_{H}$, and effective SOI field $\varphi_{{{\text{eff}}}}$ are measured from the $\left[ {1\overline{1}0} \right]$ direction (i.e., from the current direction).

**Figure 3:**
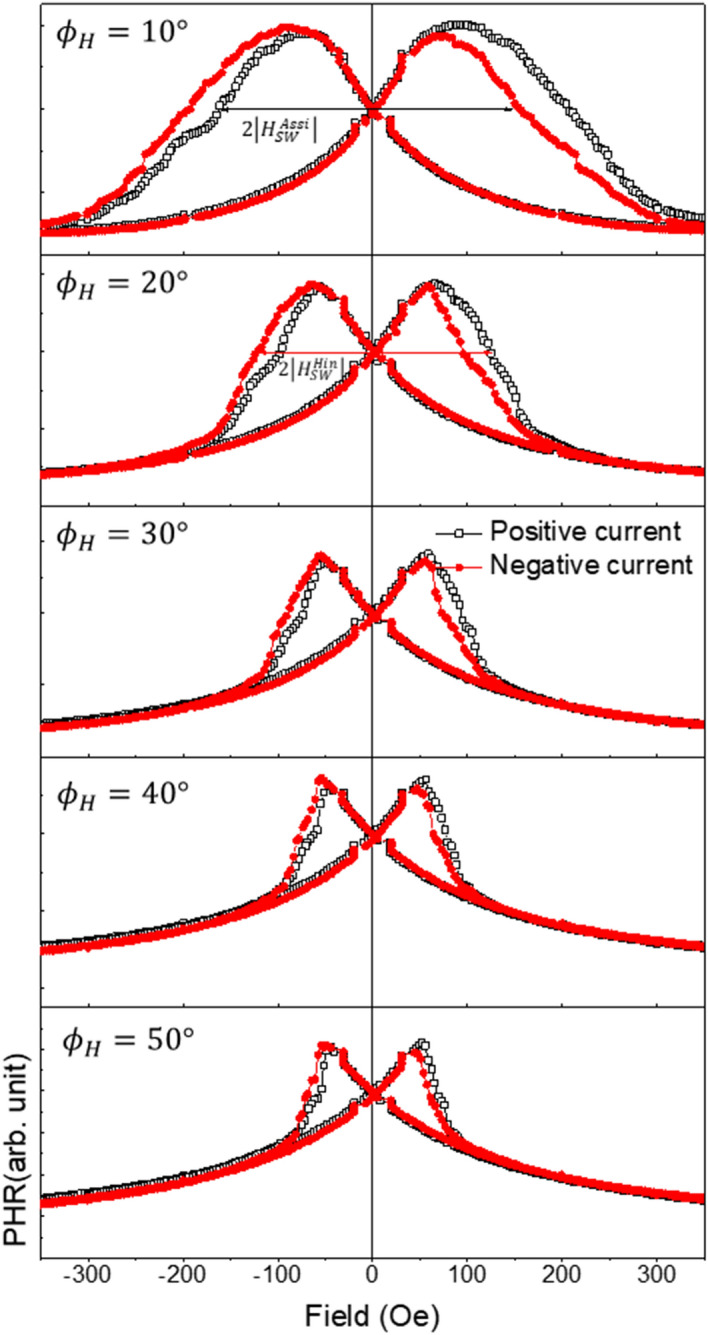
PHR data measured at 3 K with a current density of 2.5 × 10^5^ A/cm^2^, during magnetization reversal by field scan at different field directions $\varphi_{H}$. Black open and red solid symbols represent data measured when the current is positive and negative, respectively. The switching fields for $\left| {H_{SW}^{Assi} } \right|$ and $\left| {H_{SW}^{Hin} } \right|$ are marked, respectively, with black and red arrows.

The Dresselhaus-type (Rashba-type) SOI field in the GaMnAs film is at $\varphi_{{{\text{eff}}}} = - \frac{\pi }{2}$ ($\varphi_{{{\text{eff}}}} = + \frac{\pi }{2}$) when the current flows along the $\left[ {1\overline{1}0} \right]$ direction (i.e., positive current), and at $\varphi_{{{\text{eff}}}} = + \frac{\pi }{2}$ ($\varphi_{{{\text{eff}}}} = - \frac{\pi }{2}$) for the current along $\left[ {\overline{1}10} \right]$ (negative current), as shown in Fig. 1c. Since the Dresselhaus- and Rashba-type fields are always opposite to each other, with this current configuration we only measure the combined effects of these two fields (i.e., their net value). The direction of the net field is consistent with Dresselhaus-type SOI fields, as already mentioned. The presence of this $H_{{{\text{eff}}}}$ then causes the difference in the switching fields $H_{SW}^{ + I}$ and $H_{SW}^{ - I}$, that is observed with positive and negative currents, as already shown in Figs. 2 and 3. In what follows we will use this behavior for obtaining the value of the SOI field quantitatively.

In order to develop an expression for the SOI field, let us consider the CCW reorientation of magnetization over the $\left[ {\overline{1}10} \right]$ barrier (i.e., the case of transition observed in the negative field regions shown in Fig. 2). The two directions of magnetization involved in these transitions are determined by the two neighboring minima of free energy on both sides of the $\left[ {\overline{1}10} \right]$ direction. The energy difference between these minima can be obtained from Eq. (2) for the CCW rotation, as follows:

3 $$ {\Delta }E_{{\left[ {\overline{1}10} \right]}}^{{ + {\text{I CCW}}}} = E_{{\left[ {010} \right]}} - E_{{\left[ {\overline{1}00} \right]}} = 2MH_{SW}^{ + I} \sin \left( {\frac{{\varphi_{2} - \varphi_{1} }}{2}} \right){\text{sin}}\varphi_{H} - 2MH_{{{\text{eff}}}} \sin \left( {\frac{{\varphi_{2} - \varphi_{1} }}{2}} \right) $$

4 $$ {\Delta }E_{{\left[ {\overline{1}10} \right]}}^{{ - {\text{I CCW}}}} = E_{{\left[ {010} \right]}} - E_{{\left[ {\overline{1}00} \right]}} = 2MH_{SW}^{ - I} \sin \left( {\frac{{\varphi_{2} - \varphi_{1} }}{2}} \right){\text{sin}}\varphi_{H} + 2MH_{{{\text{eff}}}} \sin \left( {\frac{{\varphi_{2} - \varphi_{1} }}{2}} \right) $$

where ${\Delta }E_{{\left[ {\overline{1}10} \right]}}^{{ + {\text{I CCW}}}}$ and $\Delta E_{{\left[ {\overline{1}10} \right]}}^{{ - {\text{I CCW}}}}$ represent the domain pinning energies that occur in the CCW rotation when the current is positive and negative, respectively^28,35^; and angles $\varphi_{1}$ and $\varphi_{2}$ indicate magnetization directions in the 2nd and 3rd quadrants before and after the transition across the $\left[ {\overline{1}10} \right]$ barrier.

Equations (3) and (4) can be rearranged to obtain a relation between $H_{SW}^{ \pm I}$ and $\sin \phi_{H}$ in the form

5 $$ H_{SW}^{ + I} = A\frac{1}{{{\text{sin}}\varphi_{H} }} $$

6 $$ H_{SW}^{ - I} = {\text{B}}\frac{1}{{{\text{sin}}\varphi_{H} }} $$

where the coefficients A and B of 1/$\sin \varphi_{H}$ on the right-hand side of Eqs. (5) and (6) are

7 $$ A = \frac{{{ }\Delta E_{{\left[ {\overline{1}10} \right]}}^{ + I} }}{{2M\sin \left( {\frac{{\varphi_{2} - \varphi_{1} }}{2}} \right)}} + H_{{{\text{eff}}}} , $$

8 $$ {\text{B}} = \frac{{{ }\Delta E_{{\left[ {\overline{1}10} \right]}}^{ - I} }}{{2M\sin \left( {\frac{{\varphi_{2} - \varphi_{1} }}{2}} \right)}} - H_{{{\text{eff}}}} . $$

The values of ${\text{sin}}\varphi_{H}$ and $H_{SW}^{ \pm I}$ in Eqs. (5) and (6) can be obtained from field scans of PHR carried out at different field directions, as shown in Fig. 3.

**Figure 4:**
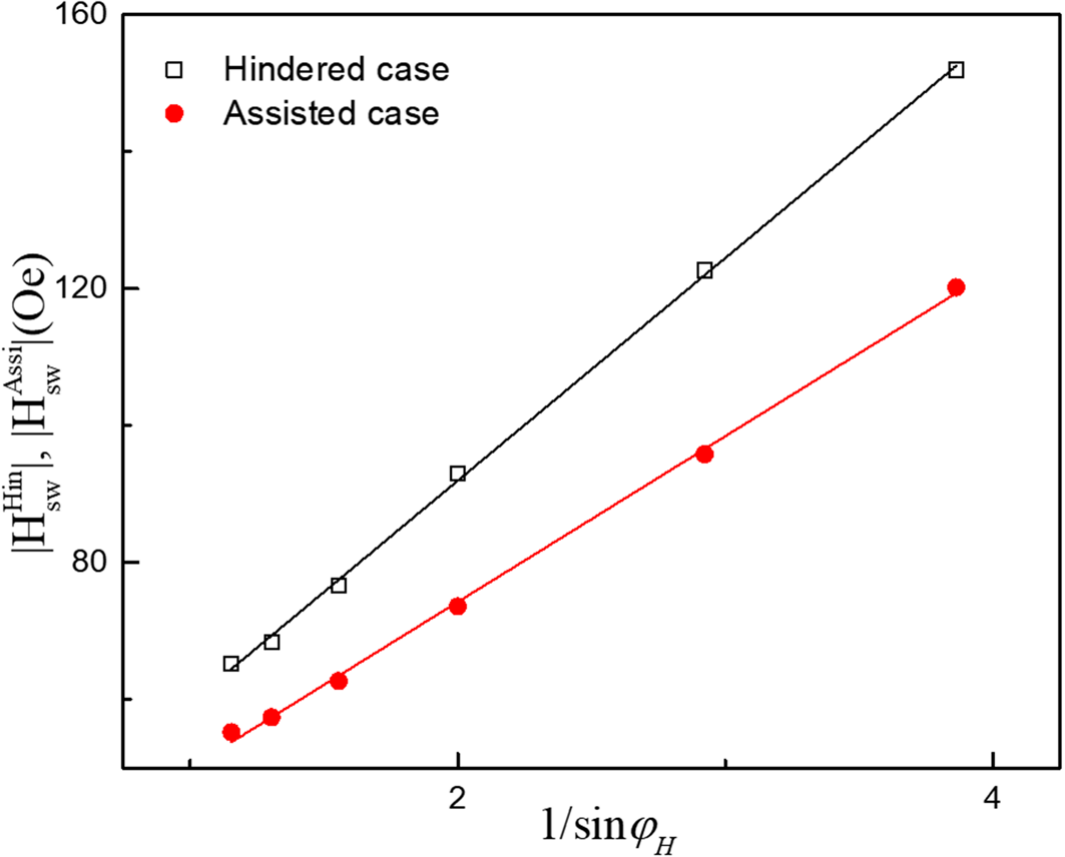
Field direction dependences of average switching fields $\left| {H_{SW}^{Hin} } \right|$ and $\left| {H_{SW}^{Assi} } \right|$ observed for the GaMnAs film with the current density of 2.5 × 10^5^ A/cm^2^. The black open squares and solid red circles represent data obtained from Fig. 3 for $\left| {H_{SW}^{Hin} } \right|$ and $\left| {H_{SW}^{Assi} } \right|$, respectively. Solid lines show fitting results obtained with Eqs. (7) and (8).

For the fitting, we use average switching fields $\left| {H_{SW}^{Hin} } \right|$ and $\left| {H_{SW}^{Assi} } \right|$ for the assisted and hindered cases, respectively, from the negative and positive field regions in Fig. 3. The values of $\left| {H_{SW}^{Hin} } \right|$ and $\left| {H_{SW}^{Assi} } \right|$ obtained at various magnetic field directions are plotted as a function of $1/{\text{sin}}\varphi_{H}$ in Fig. 4, where black open squares and red solid circles represent data for $\left| {H_{SW}^{Hin} } \right|$ and $\left| {H_{SW}^{Assi} } \right|$, respectively. Equations (5) and (6) can be fitted to the data in Fig. 4 by treating ${\text{A}}$ and ${\text{B}}$ as fitting parameters. The solid lines in the figure are best fits for the data obtained with the current density of 2.5 × 10^5^ A/cm^2^. Since the first terms on the right of Eqs. (7) and (8) are the same, the effective SOI field $H_{{{\text{eff}}}}$ can be obtained from the fitting values of A and B as ${ }H_{{{\text{eff}}}} = \left( {A - {\text{B}}} \right)/2$, giving $H_{{{\text{eff}}}} = 4.15 \pm 0.6 {\text{Oe}}$.

**Figure 5:**
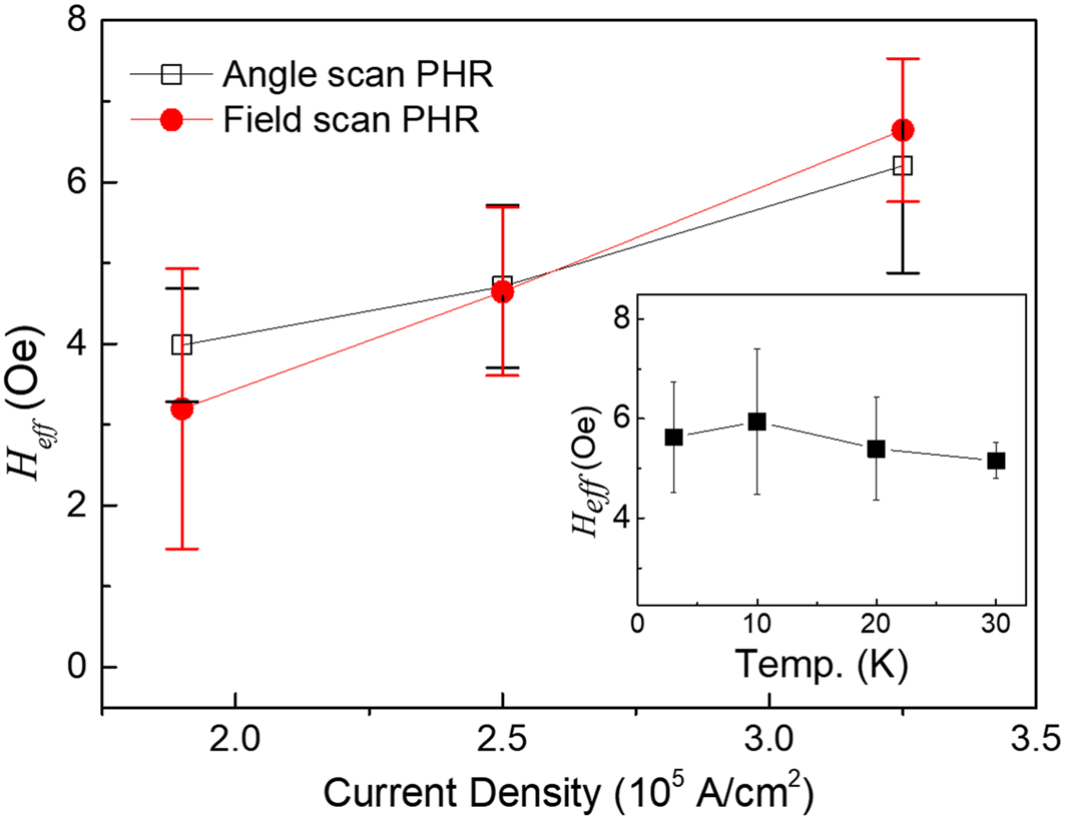
SOI fields obtained from the GaMnAs film for three different current densities. The red solid circles are obtained by analyzing the field scan PHR measurement at several different directions, as discussed in this paper. The black open symbols represent the SOI fields obtained by using methods developed in Ref^24^. Both methods provide consistent results, showing that the SOI field increases monotonically with increasing current density. Inset shows SOI fields obtained at four different temperatures for the current density of J = 2.7 × 10^5^ A/cm^2^.

We performed field-scan PHR experiments using three different current densities and analyzed them as discussed above. The SOI fields obtained from these measurements are plotted as solid circles in Fig. 5. It is clear that the value of the SO field increases linearly with current density. This trend is consistent with earlier reports in which the SOI field was obtained from the angular dependence of PHR measurements^16,17,19,20^. For direct comparison, we also carried out the angular dependent PHR measurements on our present GaMnAs film, at the same current densities as those used in the field scan PHR measurements, and obtained the values of the SOI fields following the analysis as developed in the Ref^24^. These values are plotted as open squares in Fig. 5. The two methods provide consistent results within experimental error, indicating the reliability of field scan PHR measurement to obtain the SOI field in GaMnAs films with in-plane magnetic anisotropy. Furthermore, using present method we have also investigated temperature dependence of SOI field. For this study we used a current density of J = 2.7 × 10^5^ A/cm^3^, and performed measurements at four different temperatures in the range 3–30 K. The SOI fields obtained at four different temperatures are plotted in the inset of Fig. 5. The values of the SOI field lie in the region between 5 and 6 Oe. Considering the uncertainty of the values, the SOI field does not appear to be sensitive to temperature, at least in the temperature region investigated in this study.

## Summary

In summary, we have observed differences in switching fields for opposite current polarities during magnetization reversal in GaMnAs film with in-plane magnetic easy axes that are caused by the current-dependent SOI fields in the film. In this work we have developed a method for quantitatively determining the value of the effective SOI field by analyzing the dependence of such switching fields on the current density and on the scanning field orientation. Importantly, the approach developed in this work opens the way to separate investigation of the Rashba and Dresselhaus fields in other geometries. As an example, in samples allowing for the current to flow along the [100] or [010] crystallographic directions, we can readily see from Fig. 1c that, when the scanning field is applied along the current, the difference in switching fields measures *only* the Dresselhaus-type SOI field, while only Rashba SOI field will be revealed in scanning the field perpendicular to the current. The specific configuration of our present experiment, carried out with the current along the $\left[ {1\overline{1}0} \right]$ direction, does not allow us to separate the two SOI fields. However, the understanding and the feasibility of separating Rashba- and Dresselhaus fields presented in this paper is fundamentally an important contribution to investigating spin–orbit fields and their possible applications.
